# Magnet-responsive choline carbomer ionogels as a versatile and recyclable catalyst for one-pot synthesis of benzopyran in water

**DOI:** 10.1038/s41598-023-48625-0

**Published:** 2023-12-01

**Authors:** Sara Shojaee, Najmedin Azizi, Zohreh Mirjafary, Hamid Saeidian

**Affiliations:** 1 Department of Chemistry, Science and Research Branch, Islamic Azad University, Tehran, Iran; 2 Chemistry & Chemical Engineering Research Center of Iran, P.O. Box 14335-186, Tehran, Iran; 3 Department of Science, Payame Noor University (PNU), PO Box 19395-4697, Tehran, Iran

**Keywords:** Catalysis, Green chemistry, Organic chemistry, Chemical synthesis, Chemistry

## Abstract

Ionogels are gaining popularity as a potential replacement for volatile organic solvents in various processes, such as catalysts, electrochemistry, spectroscopy, and medicinal chemistry, due to their low toxicity, high thermal stability, and good solubility. Magnet-responsive ion gels with high magnetic susceptibility are promising and can be used as catalysts, sensors, and MRI contrast agents. Herein, we fabricated simple and novel magnet choline carbomer ionogels using a precipitation-deposition method with carbomers and choline hydroxide. The morphology and structure of the resulting ionogels were analyzed using various characterization techniques, including FTIR, EDX, TGA, and SEM spectroscopy. These magnet ionogels were effective catalysts for a one-pot, three-component synthesis of benzopyran derivatives, providing mild reaction conditions, environmental friendliness, and good to excellent (78–96%) yields within a short reaction time (1–2 h). Additionally, the magnet ionogels were easily recyclable, and they could be reused up to five times without catalytic deactivation.

## Introduction

In recent years, due to their sustainability, there has been exceptional interest in using ionic liquids as an alternative to classic volatile solvents^1,2^. These media, composed of cationic and anionic components, can be designed to have specific properties owing to the reaction condition^3,4^. In this regard, the term “planner solvents” have been used to indicate the potential of this group of ecologically safe liquids for chemical reactions^5,6^. The molecular structure of ionic liquids consists of different cations and anions. Usually, the role of cation is played by a bulky organic compound (positively charged), but the anions are much smaller in volume than the cations (negatively charged), and their structure is inorganic^7,8^. Thanks to the size difference between anions and cations, the bond between the two components of ionic liquids is feeble. They do not have a crystal arrangement, so these compounds are liquid at temperatures below 100 °C^9^. Ionic liquids play a dual role as a reaction medium catalyst in electrochemistry, spectroscopy, Biology, and medicinal chemistry^10^. Ionic liquids with paramagnetic properties consist of a cation, anion, and a transition metal, mainly the Lanthanide complex systems^11^. Due to their unique physicochemical properties, magnetic ionic liquids gained innovative applications in catalysis, separation extraction, and material synthesis^12^. Since transition metals can be used in many different applications and are primarily significant catalysts, it seemed to be a good idea to turn the paramagnetic ionic liquids into magnetic ionic gels^13^. Due to the cost and recyclability limitation of pure ionic liquids, the industrial utilization of its need to be immobilized IL in a solid matrix without creating any change in the IL structure^14,15^. An ionogel combines the attributes of a solid and a liquid, displaying the properties of both IL and the solid phase except flowing^16–18^. Ionogels have recently received significant attention in transistors, batteries, supercapacitors, and fuel cells^19–21^. Ionogels can be formed in several ways and have different types of classifications. The most favorite one is their assortment, based on their solid construction. They can be organic (with a polymer), inorganic (silica-based), or organic–inorganic^22–24^.

Magnetic catalysts combined with ionic liquids represent an interesting and promising approach in the field of catalysis^25^. Applications of magnetic catalysts combined with ionic liquids can be found in various fields, including organic synthesis^26,27^, biomass conversion^28,29^, and energy-related processes^30,31^. Researchers continue to explore and develop new catalytic systems to harness the advantages offered by this combination, aiming to achieve more efficient and sustainable chemical transformations^32–35^.

Carbomers are white and fluffy powders crosslinked acrylic acid polymers employed as thickeners and rheology modifiers. They create transparent gels with a wide range of applications like personal care, home care, drugs, buffering agent, institutional care products, printing inks, adhesives, and coatings^36^. Due to their utility, reliability, biocompatibility, and rare ability to rescue doomed products, they were used for various purposes. Carbomers are acid-based polymers that are acidic in their unneutralized state and have to be neutralized with an appropriate base, such as triethanolamine, sodium hydroxide, potassium hydroxide, and EDTA, to achieve their thickening ability. We can take the benefits of the acid-based properties of such soft products to prepare the magnetic ionic liquid more straightforwardly in the presence of choline hydroxide as a gelling agent and ionic media. Choline hydroxide has been of thinking in terms of being an efficient catalyst in some chemical reactions since it is an affordable, un-contaminating, innocuous ionic liquid with basic properties which is also water-soluble^37–41^.

Recently, we reported the application of greener solvents such as water, deep eutectic solvent, and ionic liquids in various organic transformations^42–44^. Herein we report a novel and simple sol–gel method to confine the ionic liquid and magnetic nanoparticles simultaneously within a polymer matrix through a one-step process. The basic idea developed is straightforward, practical, and applicable in the industrial revolution due to its environmentally friendly nature and inexpensive starting materials.

## Experimental

### General information

All chemicals have been provided by Merck, and other available chemical suppliers and used without purification. The Buchi 53 melting point device has recorded the melting point. An illustration of FT-IR spectra was executed on a Bruker Vector-22 infrared spectrometer utilizing KBr cake and announced in cm^−1^. EDX was used for morphological studies.

### Preparation of magnetic ionogel

**Figure 1:**
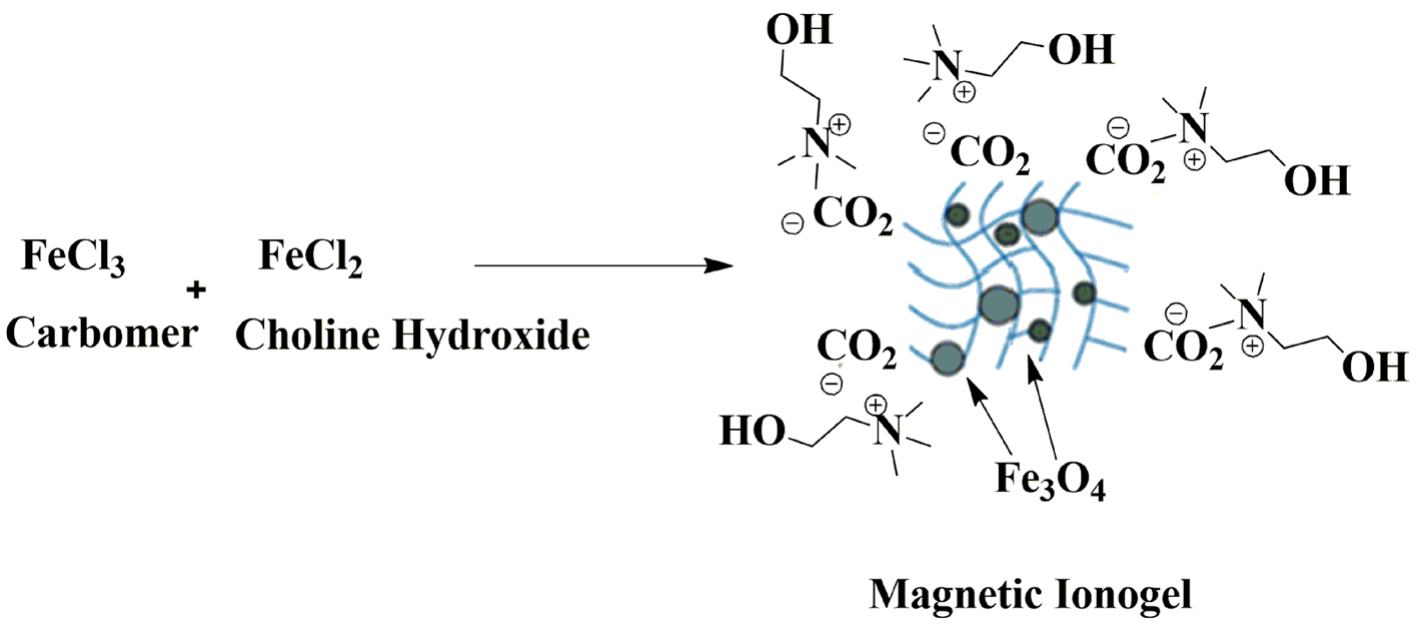
Synthesis of magnetic ionogel.

150 mL of double distilled water was poured into 500 mL of a three-neck round-bottom flask with mechanical stirring under a nitrogen atmosphere. Next, FeCl_3_·6H_2_O (8.1 g) and FeCl_2_·4H_2_O (4.97 g) were added to the flask and stirred thoroughly until the iron salts were wholly dissolved. Afterward, 2 g carbomer 940 was added to the solution under vigorous stirring at 80 °C, and the pH solution was adjusted to 10 using 40% choline hydroxide solution in water. After continuous stirring for 2 h, the magnetite precipitates were washed to pH = 7 using deionized water. The black gel was washed several times with deionized water until the pH of the eluent decreased to 7. The resulting magnetic ionogel was collected by an external magnet and washed with double distilled water until the solution was neutral. Then, it was washed with ethanol successively and dried under a vacuum (Fig. 1).

## General procedure

In a 5 mL round-bottom flasks with a magnetic stirring bar, benzaldehyde (100 µL, 1.0 mmol), dimedone (140 mg, 1.0 mmol), and malononitrile (63 µL, 1.0 mmol) in the presence of magnetic ionogel (50 mg) in water (2 mL) were added respectively. The reaction mixture was stirred at 60 °C until the reaction mixture was completed. After the reaction, ethyl acetate (10 mL) was added, and magnetic ionogel in water was separated by external magnet. After the evaporation of solvents in a rotary evaporator, the residue was recrystallized from hot ethanol to yield the corresponding pure products. All the products listed in Table 2 were synthesized following this procedure. The product yield was determined by comparing the observed melting point of the isolated product with the reported melting point range of the desired compound in the literature. This approach was used to assess the success of the synthesis and ensure the obtained product aligned with the expected properties. Furthermore, in some cases, NMR analysis was performed to assess the purity of the compounds.

Selected data:

**4a:** White solid; ^1^H NMR (500 MHz, DMSO-d6) δ 0.92 (s, 3H, Me), 1.08 (s, 3H, Me), 2.08 (d, J = 8.4 Hz, CH2, 2H), 2.24 (d, J = 8.4 Hz, CH2, 2H), 4.17 (s, CH, 1H), 7.08 (brs, NH2), 7.08–7.18 (m, 2H, Ar–H), 7.29 (t, 2H, Ar–H); ^13^C NMR (125 MHz, DMSO-d6) δ 26.9, 29.2, 32.5, 35.9, 40.2, 51.0, 59.5, 113.2, 121.0, 128.1, 127.9, 129.1, 146.1, 159.1, 162.9, 195.8;

**4b:** White solid; ^1^H NMR (500 MHz, DMSO-d6) δ 1.31 (s, Me, 3H), 1.29 (s, Me, 3H), 2.16 (d, J = 8.2 Hz, CH2, 2H), 2.35 (s, Me, 3H), 2.35 (d, J = 8.2 Hz, CH2, 2H), 4.39 (s, CH, 1H), 7.23 (brs, NH2), 7.27 (d, J = 8.1 Hz, Ar–H, 2H), 7.33 (d, J = 8.1 Hz, Ar–H, 2H); ^13^C NMR (125 MHz, DMSO-d6) δ: 22.1, 28.1, 28.9, 33.0, 36.0, 41.1, 51.2, 59.1, 114.1, 121.2, 128.0, 130.3, 137.4, 143.6, 159.2, 163.1, 195.9;

**4g:** White solid, ^1^H NMR (500 MHz, DMSO-d6) δ: 0.96 (s, Me, 3H), 1.03 (s, Me, 3H), 2.11 (d, J = 8.1 Hz, CH2, 2H), 2.29 (d, J = 8.1 Hz, CH2, 2H), 4.19 (s, 1H, CH), 7.11 (d, J = 8.9 Hz, 2H, Ar–H), 7.35 (s, 2H, NH2), 7.91 (d, J = 8.9 Hz, 2H, Ar–H); ^13^C NMR (125 MHz, DMSO-d6) δ: 28.1, 29.2, 33.1, 36.9, 51.3, 59.3, 113.4, 120.2, 129.5, 131.2, 133.4, 145.3, 159.8, 195.9.

## The possible reaction mechanism

**Figure 2:**
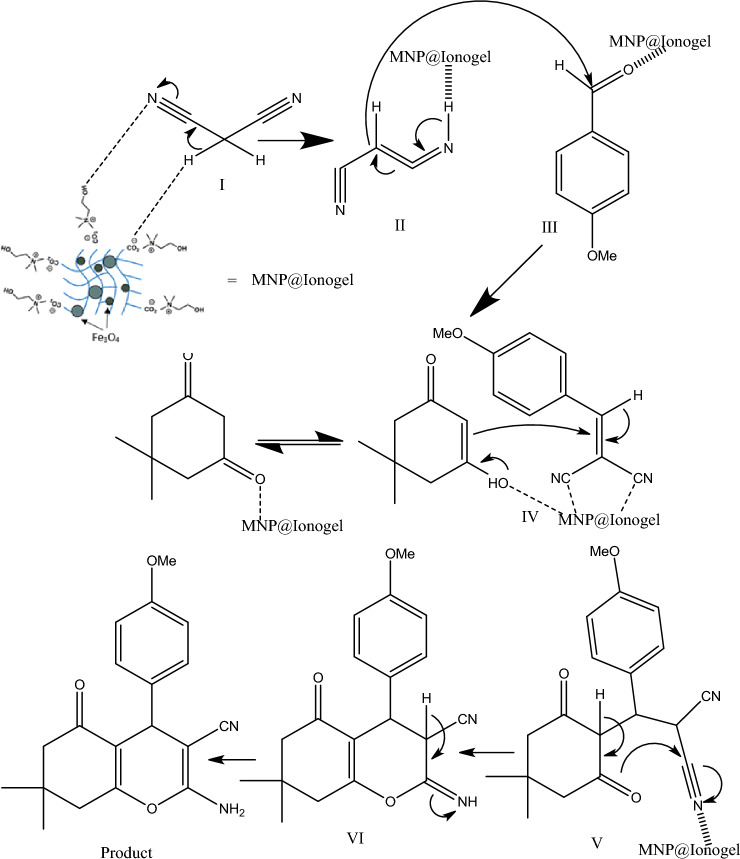
The proposed reaction mechanism.

From the possible Mechanism, we assume that the synthesized magnetic ionogel catalyst the Knoevenagel condensation reaction as well as the Micheal addition has followed. The nucleophilic addition of an activated compound to a carbonyl group (II–III intermediates) and following the reaction with the Micheal addition (Intermediate IV) of a nucleophile structure to an α, β-unsaturated carbonyl compound has led to the creation of the final product (Fig. 2).

The combination of Fe_3_O_4_ nanoparticles and choline hydroxide exhibits a synergistic catalytic effect, particularly in mild Lewis acid catalytic reactions. Fe_3_O_4_ nanoparticles possess inherent catalytic activity, acting as mild Lewis acid catalysts. On the other hand, choline hydroxide serves as a promoter or co-catalyst, further enhancing the catalytic performance of Fe_3_O_4_. The synergistic interaction between Fe_3_O_4_ and choline hydroxide facilitates surface interactions and creates an ionic medium, leading to improved reactant adsorption and promoting efficient catalytic transformations. This interaction enhances the accessibility of catalytic active sites, resulting in enhanced catalytic activity and selectivity. Moreover, the presence of Fe_3_O_4_ contributes to the stability and recyclability of the catalyst system.

## Results & discussion

### Preparation and characterization of the magnetic ionogel

**Figure 3:**
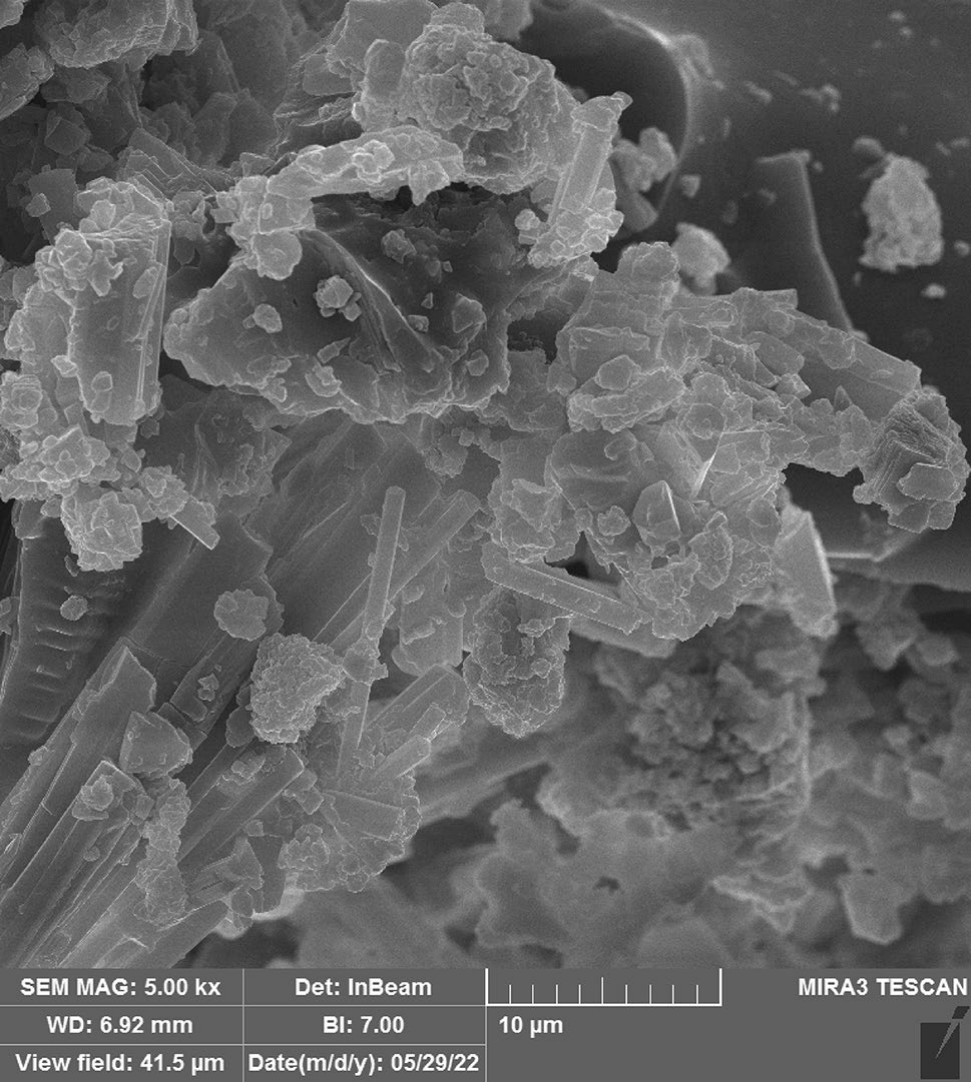
SEM image of magnetic ionogel.

The magnetic ionogel catalyst was prepared following the procedure shown in Fig. 1. The protocol design was straightforward. A reaction of choline chloride and potassium hydroxide prepared the precursor choline hydroxide. Magnetic ionogel is easily prepared via a one-pot reaction of carbomer, choline hydroxide, and iron salts in water. The morphology and distribution of the synthesized catalyst were characterized using SEM and the results were shown in Fig. 3. SEM images show the presence of some particles with different sizes formed on the carbomer surface during the process. These particles are attributed to iron oxide compositions generated under reaction conditions.

**Figure 4:**
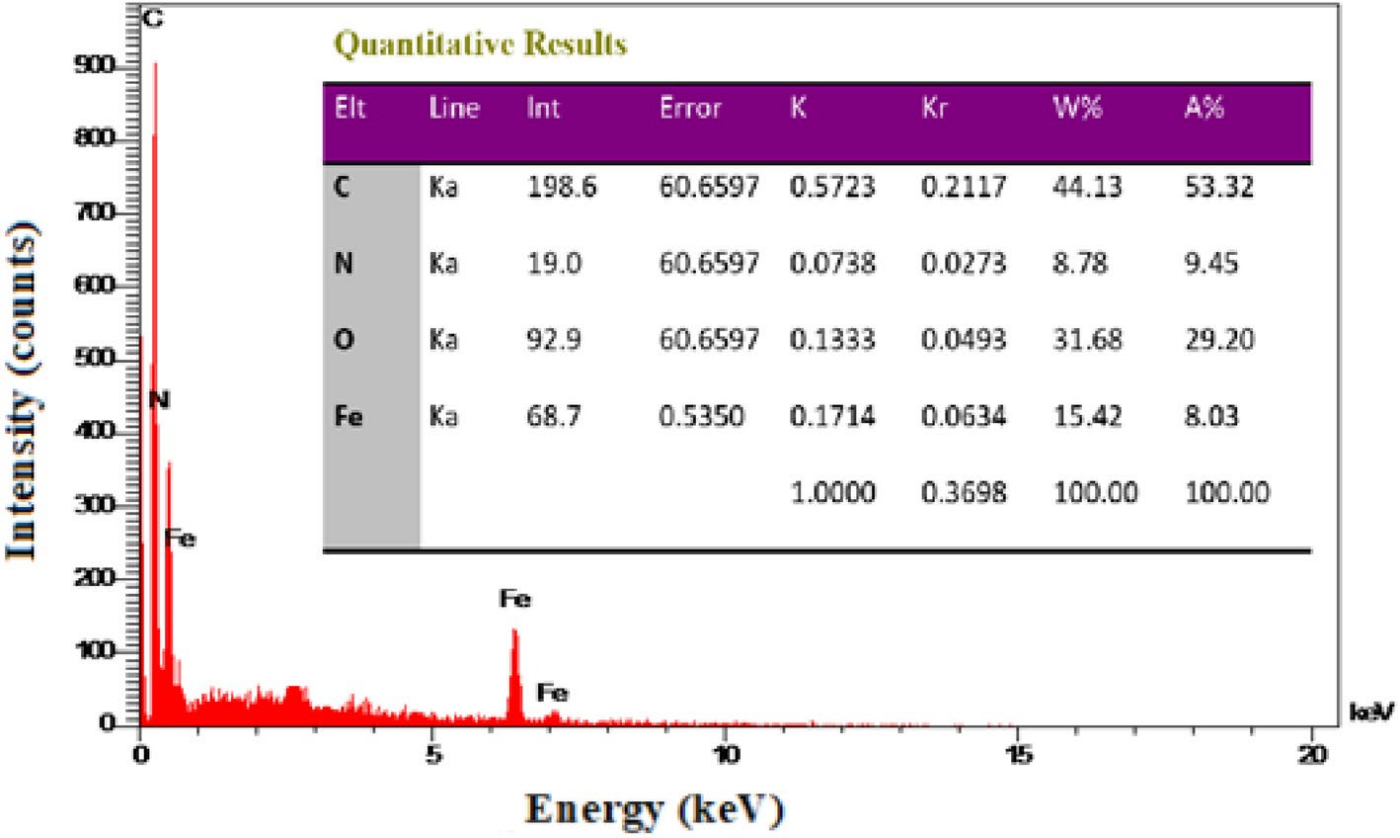
The EDX analysis of magnetic ionogel.

Energy Dispersive X-ray Spectroscopy-Mapping Analysis (EDX – MAP) was used to determine the elemental constitution of the synthesized magnetic ionogel (Fig. 4). The EDX spectrum of the catalyst shows the presence of elements C, N, O, and Fe in the catalyst, which revealed the grafting of the magnetic nanoparticles on the surface of the carbomer.

**Figure 5:**
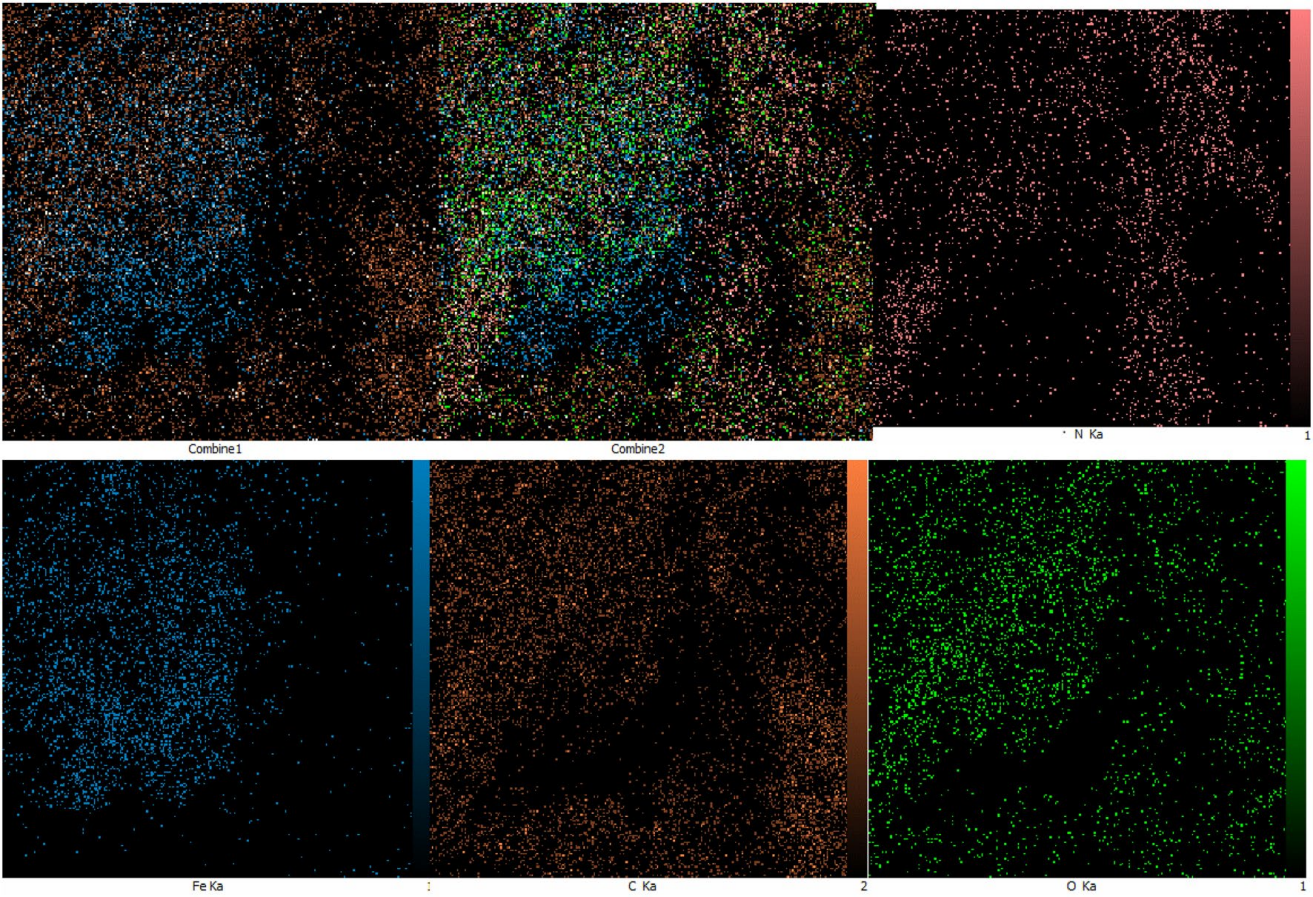
The MAP analysis of magnetic ionogel.

The MAP analysis demonstrated the frequency distribution of the elements of C, O, N, and Fe and their distribution in the magnetic ionogel, as shown in Fig. 5.

**Figure 6:**
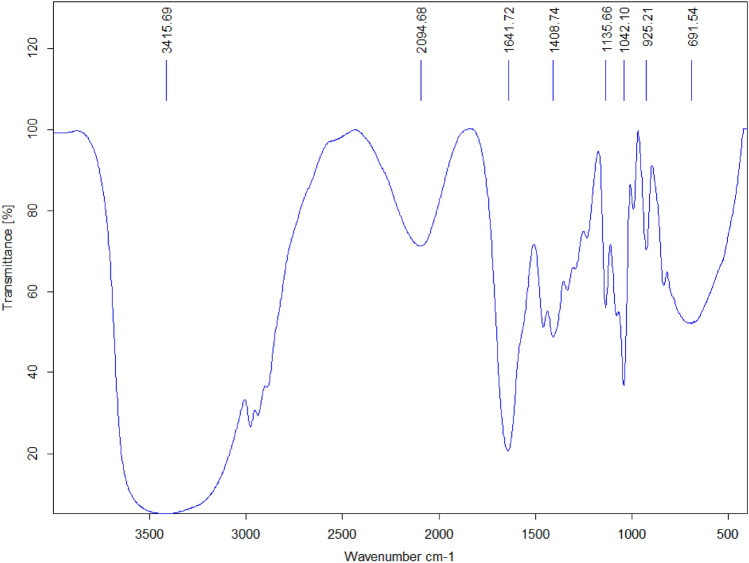
The FT-IR analysis of magnetic ionogel.

The detailed investigation of the functionalization of magnetic ionogel was performed by FT-IR spectroscopy (Fig. 6). In accordance with the result of FT-IR analysis of the carbomer based magnetic ionogel, a peak in 696 cm^–1^, can be associated to the stretching of Fe–O that is related to Fe_3_O_4_ particles which have been trapped in the gel structure. The strong band observed at around 1042 cm^–1^ can be demonstrated for the C–N stretching bond, while 1066 cm^–1^ is for the C–O bond. On the other hand, the band at 1408 cm^–1^ stands for the symmetrical stretching bond of C–O, and 1451.8 cm^–1^ can show CH_2_ bending bond. In addition, 1641 cm^–1^ illustrates C=O asymmetric bond in carbomer. The catalyst exhibit adsorptions at 2956 cm^–1^ C–H stretching vibration of carbomer followed by 3415 cm^–1^ and 2049 cm^–1^ due to the O–H groups water in the gel matrix.

**Figure 7:**
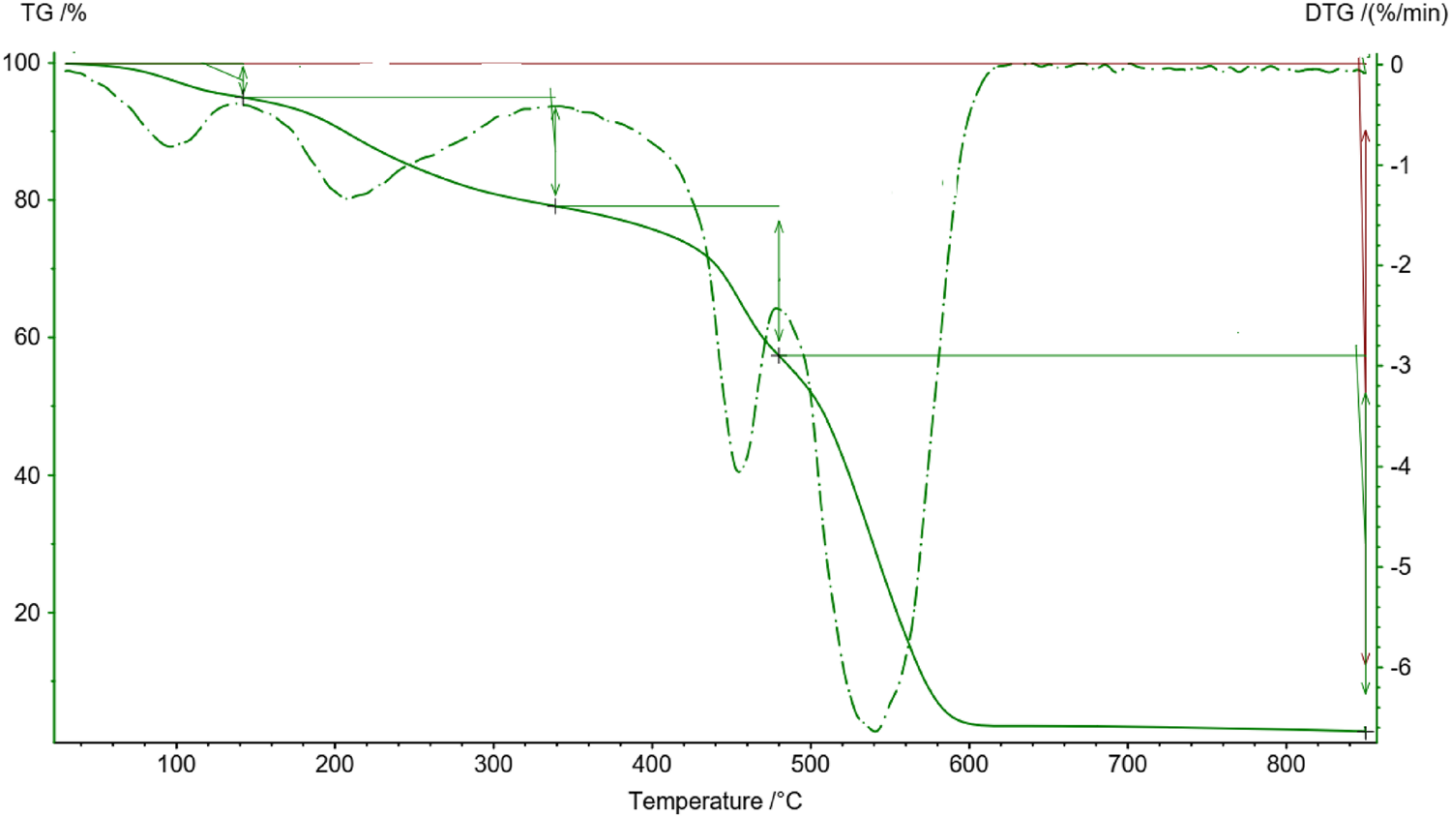
The TGA analysis of magnetic ionogel.

Thermogravimetric analysis (TGA) is a powerful analytical technique used to investigate the thermal stability of materials by measuring their weight changes as a function of temperature or time under controlled conditions. TGA analysis is used to study the thermal properties of magnetic Fe_3_O_4_-supported ionogels, composite materials composed of Fe_3_O_4_ nanoparticles, and ionic liquids immobilized in a solid matrix (Fig. 7). The TGA analysis of magnetic ionogel typically reveals two main stages of weight loss. The first stage corresponds to the removal of adsorbed water and other volatile components, while the second stage involves the degradation of the ionic liquid and the organic matrix.

**Figure 8:**
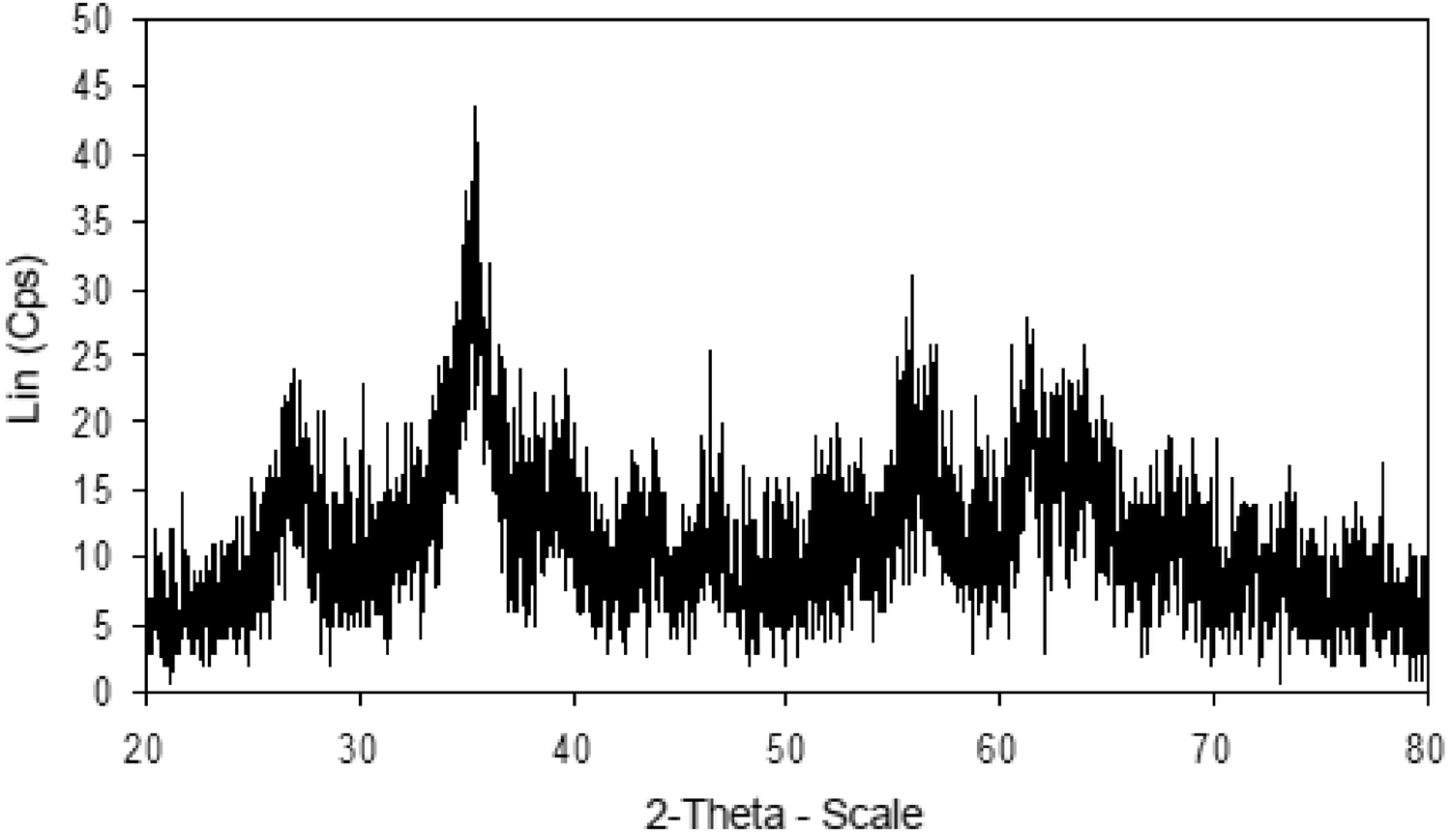
The XRD pattern of magnetic ionogel.

The structure of the magnetic ionogel was characterized by X-ray diffraction (XRD), and the corresponding patterns are shown in Fig. 8. The XRD analysis revealed characteristic diffraction peaks which can be attributed to the (220), (222), (400), (511), (440), and (533) crystal planes, respectively. These peaks are in good agreement with the standard Fe_3_O_4_ nanoparticles (PDF# 88-0315). However, the XRD patterns of the magnetic gel showed exhibit broader or less intense diffraction peaks, indicating a disruption or distortion in the crystalline structure. This can be attributed to the presence of gel matrix in the surface Fe_3_O_4_.

The catalytic activities of magnetic ionogel were investigated using the one-pot, three-component condensation reaction between benzaldehydes (1 mmol), dimedone (1mmol), and malononitrile (1 mmol) in deionized water (2.0 mL) as the model reaction (Table 1). According to the results provided in Table 1, the magnetic ionogel performed well to give the desired product within 4 h in 68% yield at room temperature (Table 1, entry 1). As we expected, the outcome of the desired products could be improved to 96% within 1 h by increasing the reaction temperature to 60 °C (Table 1, entry 3). The model reaction was carried out in various polar and nonpolar solvents (Table 1, entries 5–10) to investigate the solvent effect. The model reaction in organic solvents such as toluene, ethyl acetate, CH_3_CN dimethylformamide, and tetrahydrofuran was unsuccessful in the presence of magnetic ionogel. Next, the loaded amounts of magnetic ionogel were investigated. Increasing the catalyst to 100 mg, did not change the reaction conditions in terms of yields and times (Table 1, entry 15). On the other hand, the amount of catalyst is reduced to 10 mg, and increased reaction time was required to get the optimal results (Table 1, entries 11–14).

**Table 1:** Optimization of model reaction in the presence of magnetic ionogel.


Entry	Magnetic ionogel (mg)	Solvents (2 mL)	Time (h)	Temp. (°C)	Yields (%)^a^
1	50	Water	4	rt	68
2	50	Water	4	40	75
3	50	Water	1	60	96
4	50	Water	1	80	96
5	50	Ethanol	1	60	83
6	50	DMF	1	60	67
7	50	THF	1	60	56
8	50	Toluene	1	60	45
9	50	Ethyl acetate	1	60	48
10	50	CH_3_CN	1	60	54
11	40	Water	1	60	92
12	30	Water	1	60	90
13	20	Water	2	60	78
14	10	Water	2	60	68
15	100	Water	1	60	96

^a^Isolated yields.

Temperature plays a crucial role in catalytic reactions, impacting reaction kinetics, thermodynamics, and the stability of intermediates and transition states. In the Fe_3_O_4_ and choline hydroxide ionogel catalytic system, the temperature dependence of the product yield can be attributed to factors such as reaction rate and equilibrium position. In the specific case of this system, it has been observed that after 24 h of reaction, only the reaction between aldehydes and malononitrile was observed, and the desired benzopyran products did not show improved yields. However, it is important to consider the influence of the equilibrium position on the overall product yield. The cyclization reaction leading to benzopyran products may be reversible, and the equilibrium position can be temperature-dependent. By increasing the temperature, it becomes possible to shift the equilibrium towards the desired benzopyran products, thereby increasing their yields.

To study the green protocol's scope and generality, a series of 4H-pyran derivatives were synthesized from various aldehydes with different electronic properties (Table 2) under optimal conditions. All the tested aldehydes afforded the corresponding 4*H*-pyran in good to excellent yields. The reaction worked well with aromatic aldehydes containing electron-withdrawing groups or electron-donating to give the desired products good to excellent yields with high purity. In addition, our environmentally friendly strategies are successful for more challenging acid-sensitive aldehydes such as 2-furfural that proceed with smooth condensation without forming any side products. (Table 2, entry 12). The one-pot, three-component cyclization reaction proceeded smoothly in water and was completed in 1 to 2 h.

**Table 2:**
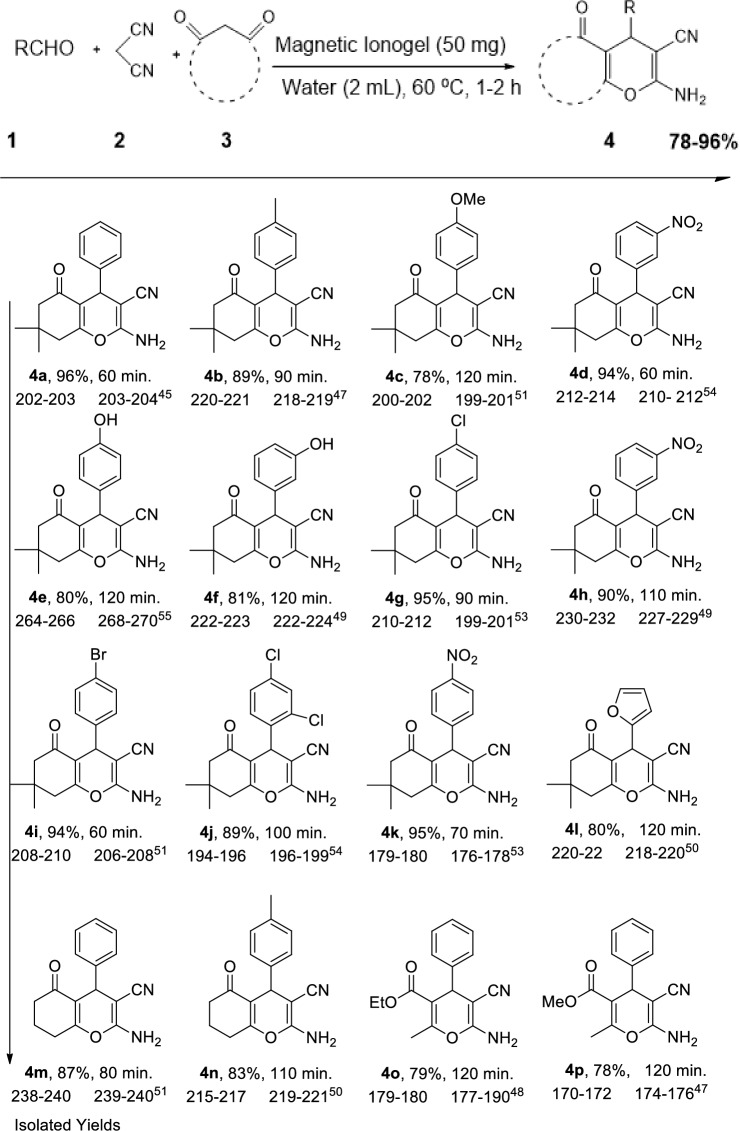
Synthesis of 4*H*-pyran derivatives with the magnetic ionogel catalyst in water.

**Figure 9:**
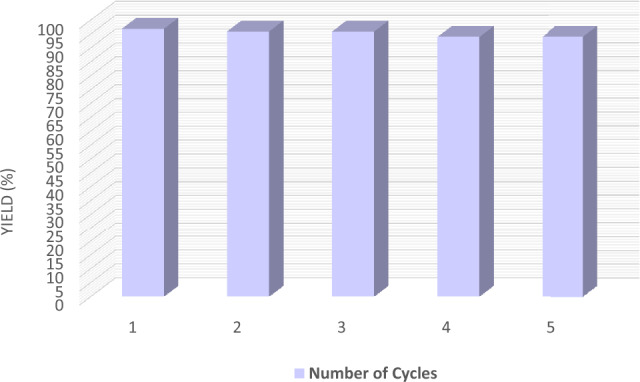
The reusability study of magnetic ionogel.

In addition to high catalytic activity and inexpensive properties of magnetic ionogel, simple separation is another advantage in industrial processes. The magnetic ionogel could be recycled easily. The one-pot reaction of benzaldehyde, dimedone, and malononitrile was chosen as a model reaction. The yields corresponding to five consecutive runs are shown in Fig. 9. After each cycle, ethyl acetate was added to the reaction mixture, and products and unreacted starting materials were recovered. The aqueous solution containing magnetic ionogel was reused for the next run with good reusable stability under air without apparent deactivation. The TON (Turnover Number) and TOF (Turnover Frequency) values for the five-cycle run using a reused catalyst were determined to be 2802 and 56 h^–1^, respectively.

**Figure 10:**
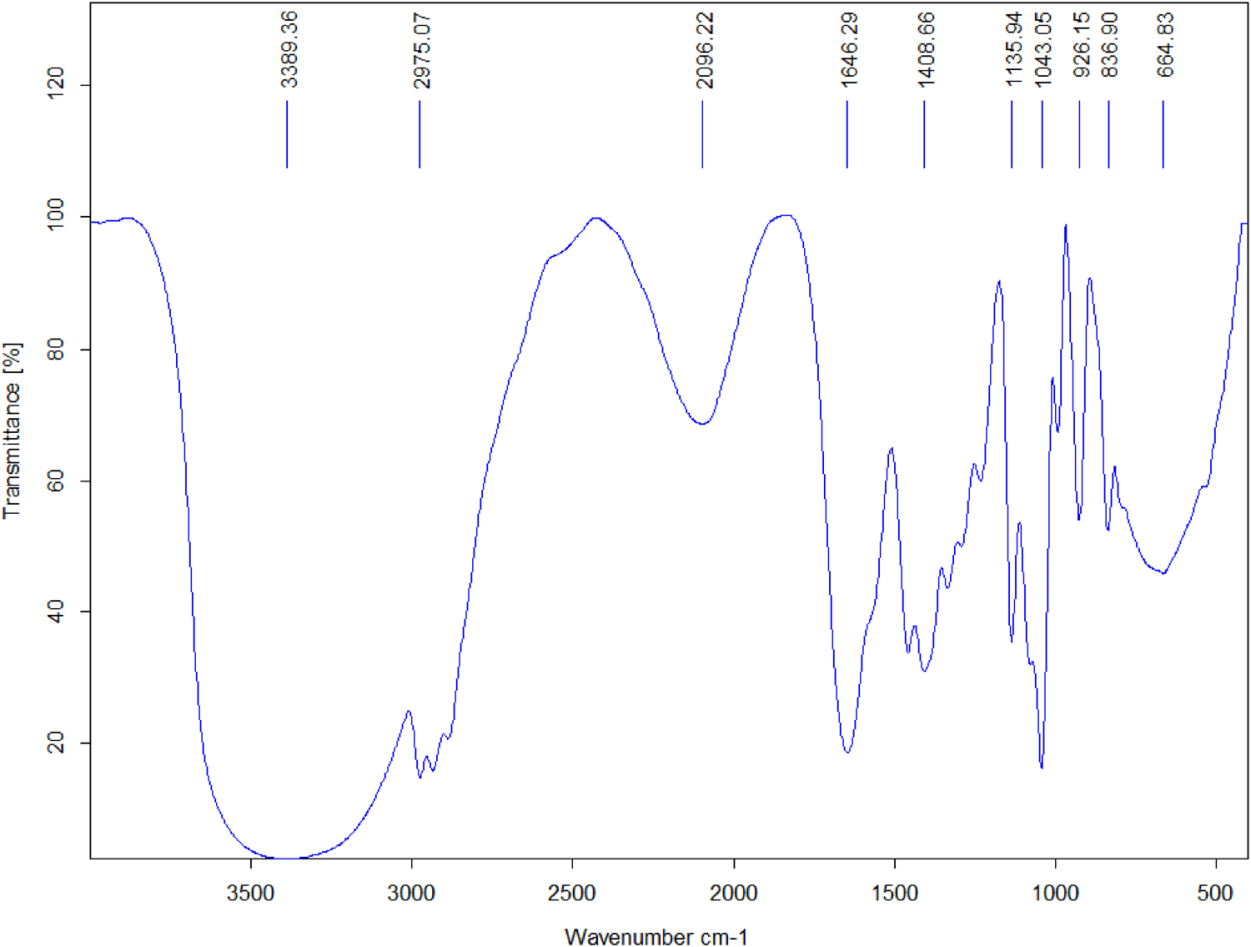
FTIR spectra of reused magnetic ionogel.

**Figure 11:**
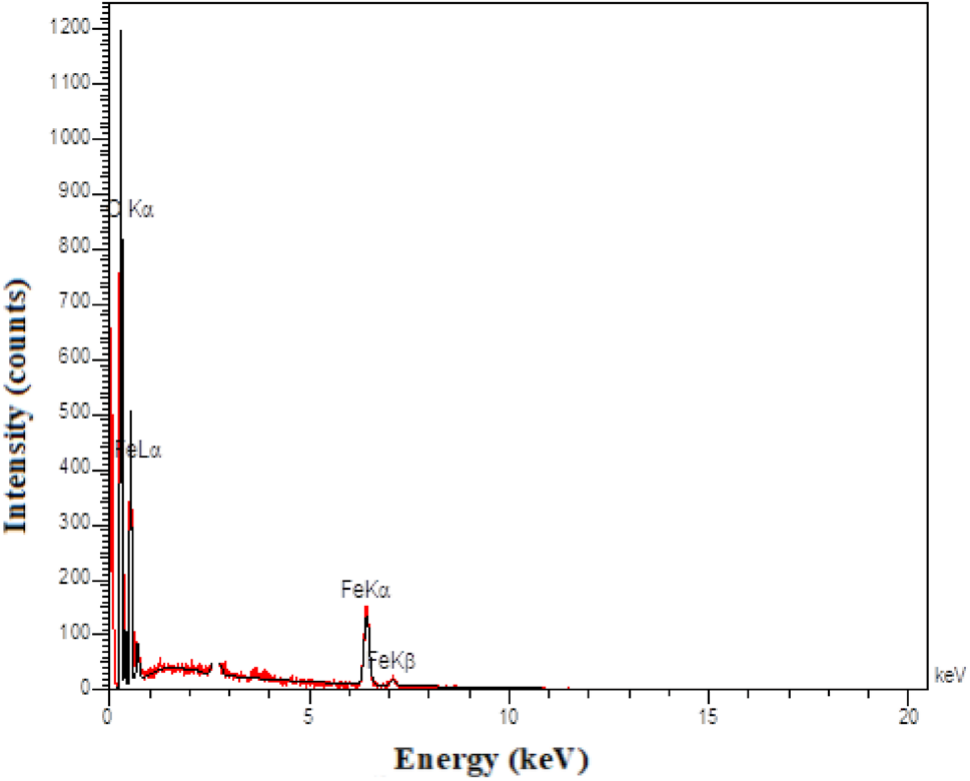
EDS image of reused ionogel.

Analyzing the reused ionogel using FTIR (Fig. 10) and EDX spectrometry (Fig. 11) is a common approach to assess the stability and changes in catalysts. The fact that the results showed no change after the five cycle run indicates that the catalyst remained stable throughout the experimental process.

Table 3 showed the comparison of the activity of magnetic ionogels with various methods commonly used for the one-pot synthesis of benzopyran derivatives in the literature. Magnetic ionogels offer a promising approach for the one-pot synthesis of benzopyran derivatives^45–56^. They combine the advantages of magnetic nanoparticles and ionic liquids or ionogels, providing improved catalytic activity, easy separation, and recyclability (Table 3).

**Table 3:** A comparison of the activity of magnetic ionogels with various reported procedure for the one-pot synthesis of benzopyran derivatives (**4a**).

Method	Reaction conditions	Yield (%)	Reusability	Refs.
A	Neat, 100 °C; 1h; Basic conditions	100	1	^45^
B	HAHS’ ionic liquid, 0.75 h	99	1	^46^
C	Chitosan Based Heterogeneous Catalyses: ethanol: 3 h	95	1	^47^
D	Lithium perchlorate In acetonitrile at 20 °C ; 3 h; Electrolysis;	92	1	^48^
E	Triethylamine In water at 50 °C; Microwave irradiation;	92	1	^49^
F	Silica gel for 0.0333333 h; microwave irradiation;	91	1	^50^
G	Amine In N,N-dimethyl-formamide at 120 °C ; microwave irradiation;	79	1	^51^
H	Meglumine In ethanol; water at 20 °C; for 0.0833333 h;	97	1	^57^
I	Zinc(II) oxide In ethanol; water at 20 °C ; 3 h;	95	3	^52^
J	Ferrocene-Functionalized Dithiocarbamate Zinc In ethanol at 20 °C ; for 2 h;	95	1	^53^
K	Magnetically tuned halloysite functionalized sulfonic acid In ethanol; water at 80 °C ; for 1.5 h;	95	5	^54^
L	Pyridine N-oxide; silver(l) oxide In ethanol at 70 °C; for 1.33333 h;		1	^55^
M	Cobalt ferrite In ethanol; water for 0.116667 h;	93	5	^56^
N	Magnetic ionogel, water at 60 °C; for 1.5 h	97	5	This work

## Conclusion

The study presents a methodology for synthesizing benzopyran derivatives using a magnetic ionogel catalyst in water, which offers several advantages over traditional methods. The development of the magnetic ionogel catalyst was achieved using a simple and novel approach, resulting in a highly efficient catalyst that led to the formation of a variety of benzopyran compounds with excellent yields in a short reaction time. Furthermore, the catalyst was easily recoverable, and the reaction system could be reused for up to five cycles with consistent yields. These results highlight the potential of magnetic ionogels as sustainable and versatile catalysts for various chemical transformations. This work contributes to the field by introducing a novel approach to fabricating magnet choline carbomer ionogels for the first time and showcasing their potential as multifunctional materials with applications in catalysis, sensing, and medical imaging.

## Data Availability

The data that support the findings of this study are available on request from the corresponding author.
